# Evaluation of the pro-healing effect of a commercial formulation of aqueous extract of *Triticum vulgare* in hyperglycemic mice

**DOI:** 10.7705/biomedica.7976

**Published:** 2026-08-20

**Authors:** Omar Casadiego, Fernando Rodríguez-Sanabria, Guillermina Baay-Guzmán, Julio César Mantilla, Patricia Escobar

**Affiliations:** 1 Centro de Investigaciones en Enfermedades Tropicales, Departamento de Ciencias Básicas, Escuela de Medicina, Universidad Industrial de Santander, Piedecuesta, Colombia Universidad Industrial de Santander Escuela de Medicina Universidad Industrial de Santander Piedecuesta Colombia; 2 Unidad de Investigación en Enfermedades Oncológicas, Hospital Infantil de México Federico Gómez, México, D. F., México Hospital Infantil de México Federico Gómez México D. F México; 3 Departamento de Patología, Escuela de Medicina, Universidad Industrial de Santander, Bucaramanga, Colombia Universidad Industrial de Santander Escuela de Medicina Universidad Industrial de Santander Bucaramanga Colombia

**Keywords:** Wound healing, diabetes mellitus, Triticum, hyperglycemia, streptozotocin, cicatrización de heridas, diabetes mellitus, Triticum, hiperglucemia, estreptozotocina

## Abstract

**Introduction.:**

*Triticum vulgare* extract, marketed as Fitostimoline™, is used to stimulate re-epithelialization and tissue regeneration. The use of this extract in chronic ulcers such as diabetic foot ulcers may represent a viable treatment alternative.

**Objective.:**

To study the effects of 15% *T. vulgare* extract cream on wound healing in normoglycemic and streptozotocin-induced hyperglycemic BALB/c mice.

**Materials and methods.:**

Hyperglycemia was induced in mice with a single dose of streptozotocin (200 mg/kg) (group STZ-1) or with multiple doses (30 mg/kg for five consecutive days) (group STZ-2). Excisional wounds were created on the dorsal skin, and *T. vulgare* extract was applied topically twice daily for ten days. We assessed wound closure, histological changes, and inflammatory markers.

**Results.:**

*Triticum vulgare* extract treatment significantly enhanced wound closure in both normoglycemic and hyperglycemic mice compared to controls. Histological analysis revealed fewer mast cells, reduced inflammation in *T. vulgare* extract-treated groups, and increased collagen deposition in the treated normoglycemic and STZT-1 groups. *Triticum vulgare* extract treatment also reduced IL-6 and IL-1 ß levels in certain hyperglycemic groups.

**Conclusions.:**

Overall, in this murine model, the topical application of *T. vulgare* extract improved wound healing in both normoglycemic and hyperglycemic conditions by accelerating closure, improving histological architecture, and rebalancing inflammatory cytokines toward a pro-healing profile, indicating its potential as a therapeutic option for diabetic wound care. The findings suggest multiple mechanisms of action for *T. vulgare* extract. Nonetheless, further research is necessary to elucidate these mechanisms and to fully explore potential clinical applications.

## Resumen

**Introducción.:**

El extracto de *Triticum vulgare,* comercializado como Fitostimolina™, se utiliza en los casos que requieren estimulación de los procesos de reepitelización o regeneración de tejidos. Su aplicación en úlceras crónicas, como las del pie diabético, podría constituir una alternativa terapéutica.

**Objetivo.:**

Evaluar los efectos del extracto de *T. vulgare* al 15 %, en crema, para la cicatrización de heridas de ratones BALB/c normoglucémicos o hiperglucémicos.

**Materiales y métodos.:**

La hiperglucemia se indujo con una dosis de 200 mg/kg de estreptozotocina (grupo STZ-1) o con múltiples dosis de 30 mg/kg (grupo STZ-2) durante cinco días consecutivos. Se causaron heridas por escisión de la piel del dorso de los ratones y se aplicó crema del extracto de *T. vulgare* dos veces al día durante 10 días. Se evaluaron el cierre de la herida, los cambios histológicos y los marcadores inflamatorios.

**Resultados.:**

El tratamiento con extracto de *T. vulgare* mejoró significativamente el cierre de la herida en los ratones normoglucémicos e hiperglucémicos en comparación con el grupo control no tratado. En el análisis histológico de los ratones tratados con extracto de *T. vulgare* se encontró un menor número de mastocitos, una reducción de la inflamación y un mayor depósito de colágeno en el grupo normoglucémico tratado y en el grupo STZT-1. El tratamiento con extracto de *T. vulgare* también disminuyó los niveles de IL-6 e IL-1 ß en los dos grupos de ratones.

**Conclusiones.:**

En conjunto, la aplicación tópica del extracto de *T. vulgare* en este modelo en ratones mejoró la cicatrización en condiciones normoglucémicas e hiperglucémicas al acelerar el cierre, optimizar la arquitectura histológica y reequilibrar las citocinas inflamatorias hacia un perfil procicatrizante, lo cual indica su potencial como opción terapéutica para el cuidado de las lesiones por diabetes. Los hallazgos sugieren múltiples mecanismos de acción del extracto de *T. vulgare.* No obstante, se requieren investigaciones adicionales para dilucidar estos mecanismos y explorar plenamente sus posibles aplicaciones clínicas.

Diabetes mellitus comprises a group of metabolic disorders marked by elevated blood glucose levels, which adversely affect the quality of life and lead to chronic complications involving various organs. Among patients with diabetes mellitus, there is a considerable risk of developing diabetic foot ulcers, a severe condition that can lead to limb amputation in nearly 50% of cases ^1^. A diabetic foot ulcer develops in 19-34% of diabetes mellitus patients, with about 20% needing a minor or major lower-extremity amputation ^2^.

Skin healing typically proceeds through three phases: inflammation, proliferation, and remodeling. Initially, a fibrin clot forms, creating a scaffold for immune cells to clear away pathogens and debris. This clot also releases cytokines and growth factors that initiate the healing process. During the proliferation phase, epithelial cells migrate to cover the wound, new capillaries develop, and fibroblasts produce collagen, forming granulation tissue. In the final remodeling phase, collagen fibers are reorganized to enhance tensile strength, the extracellular matrix is remodeled, and cell number is reduced through apoptosis. This process ultimately restores tissue integrity ^3^.

In diabetic patients, ischemia, hypoxia, and a high glucose environment impair the wound-healing process, leading to delayed or non-healing wounds ^4^. Hyperglycemia contributes to arterial stiffness, reduced blood flow, and peripheral neuropathy. This reduces pain sensitivity, making it difficult to detect and treat wounds.

Inflammation results from the sustained release of cytokines such as interleukin (IL) 1, IL-6, and tumor necrosis factor-alpha (TNF-α), along with increased reactive oxygen species ^5^^,^^6^. Additionally, there is a decrease in vascular endothelial growth factor (VEGF) expression, thereby affecting tissue angiogenesis ^7^. Recruitment of keratinocytes, fibroblasts, and endothelial cells, as well as angiogenesis and extracellular matrix synthesis, is impaired by low production of growth factors such as insulin-like growth factor 1 (IGF-1) and transforming growth factor-ß (TGF-ß) ^8^.

Treating chronic wounds includes debridement, dressings, growth factors, cell therapy, and management of the underlying disease ^9^. Natural plant-derived compounds are being investigated as potential treatments due to their antiinflammatory, antioxidant, and antimicrobial properties, as well as their ability to promote cell proliferation, extracellular matrix formation, and angiogenesis ^10^^-^^12^.

*Triticum vulgare* (common wheat) is a member of the Graminaceae family, traditionally used in medicine to promote tissue repair ^13^^,^^14^. The extract of *T. vulgare* derived from the entire germinated plant primarily contains polysaccharides and oligosaccharides. Patented by Farmaceutici Damor, it is the active agent in various medical devices marketed under the trade name Fitostimoline™. These products are widely used in tissue repairs due to their capacity to promote fibroblast and endothelial cell proliferation and to reduce inflammation ^13^^-^^16^.

Streptozotocin, extracted from the fungus *Streptomyces achromogenes,* is used to destroy pancreatic ß-cells in rodents, creating a model of diabetes mellitus with symptoms such as polydipsia and polyuria ^17^^,^^18^. This method is widely used because the murine model offers multiple protocols for inducing hyperglycemia. The multiple low-dose model is the most used, as it closely mimics the pathogenesis of type 1 diabetes mellitus ^19^.

This study aimed to assess the effect of Fitostimoline™, a commercially available *T. vulgare* extract, on the healing of excisional wounds in normoglycemic and streptozotocin-induced hyperglycemic BALB/c mice, using single- or multiple-dose induction protocols.

## Materials and methods

### Reagents and compounds

Sodium citrate, citric acid, and streptozotocin were purchased from Sigma-Aldrich (St. Louis, USA). Xylazine and ketamine were commercially obtained from Erma (Colombia), and Chemiesa (Chile), respectively. Formaldehyde was acquired from Inquimicol (Colombia). Streptozotocin doses were prepared in a fresh 0.1 M citrate buffer (pH 4.5) immediately before application. Fitostimoline™ cream (Rigenase™, 15% w/w aqueous extract of *T. vulgare;* lot L:3109/2019; exp. 06/2021) was obtained from EuroEtika (Colombia).

### Animals and ethical considerations

BALB/c mice aged 10 to 12 weeks were obtained from the *Instituto Nacional de Salud* of Colombia animal facility in Bogotá. They were maintained on a 12-hour light/dark cycle at 23°C with 55% relative humidity and had access to food and water *ad libitum.* Animal welfare was prioritized throughout the study. Animal procedures were performed in accordance with the recommendations outlined in the *2011 NIH guide for the care and use of laboratory animals* (https://www.ncbi.nlm.nih.gov/books/NBK54050/), published by the National Research Council (US) Committee. The Scientific Research Ethics Committee of the *Universidad Industrial de Santander* approved the study, recorded under Act No. 4110 on March 6, 2020.

### Treatment with streptozotocin and glycemia monitoring

Streptozotocin was administered by intraperitoneal injection, and blood glucose levels were monitored using a rapid test with reactive strips and a Contour XT glucometer (Bayer, North Rhine-Westphalia, Germany). Two dosing regimens were used: a single dose of 200 mg/kg (STZ-1 group, n = 7) and daily doses of 30 mg/kg for five days (STZ-2 group, n = 7). Blood samples were collected from the tail vein, and glucose levels were measured prior to streptozotocin administration and monitored weekly. Mice with glucose levels exceeding 250 mg/dl and maintained for two weeks were classified as hyperglycemic ^20^.

### Wound healing assay

*Wound model.* The study used the excisional wound model in BALB/c mice ^21^. In hyperglycemic mice, excisional wounds were made one week after hyperglycemia was confirmed. The mice were shaved, and 24 hours later, two excisional wounds were made on either side of the dorsal midline using a 5 mm surgical punch under anesthesia via intraperitoneal injection of a ketamine/xylazine cocktail.

*Experimental design.* The mice were randomly divided into six groups, each containing seven mice (table 1). The sample size (80% power, 0.05 alpha) was calculated based on a pilot test (n = 3) using G*Power, version 3.1.9 (Universität Düsseldorf). Topical *T. vulgare* extract (~100 mg per excision) was applied to the treated mice twice daily for 10 days. Mice were observed for one hour after treatment application to prevent ingestion. Some groups remained untreated (table 1).

**Table 1:** Experimental groups

	Group	n	Code	Streptozotocin	Fitostimoline™
Normoglycemic	1	7	CG	Untreated	Untreated
	2	7	CG**T**	Untreated	2 times a day for 10 days
Hyperglycemic	3	7	STZ-1	200 mg/kg x 1 day	Untreated
	4	7	STZ**T**-1	200 mg/kg x 1 day	2 times day for 10 days
	5	7	STZ-2	30 mg/kg x 5 days	Untreated
	6	7	STZ**T**-2	30 mg/kg x 5 days	2 times a day for 10 days

CG: control group; CGT: control group treated with Fitostimoline™, STZ-1: single dose of streptozotocin; STZ-2: multiple doses of streptozotocin, STZT-1 and STZT-2: hyperglycemic mice treated with Fitostimoline™

*Wound closure analysis.* Photographic images of the wounds were captured using a digital camera on days 1, 3, 5, 8 and 10. The wound area was measured from these photographic records using ImageJ software (NIH), with a 1 x 2 cm reference rectangle. Wound area measurements were taken at each time point, and the percentage of wound closure was calculated using the formula:

% W closure = (initial W - final W) / initial W * 100.

To summarize overall healing, we computed the area under the curve (AUC) of open wound area versus time over the 10-day observation period (units: mm^2^ per day). For each mouse, the wound area at each time point was integrated using the trapezoidal rule in GraphPad Prism. Lower AUC values indicate faster and more complete wound closure, whereas higher AUC values reflect delayed healing ^22^.

### Histological analysis

The mice were sacrificed 6 hours after the end of treatment. Biopsies were taken from the wounds, fixed in 10% buffered formalin for 24 hours, dehydrated, embedded in paraffin, and sectioned at 5 urn using a microtome. Dewaxed slides were stained with hematoxylin and eosin, toluidine blue and Masson's trichrome, and processed ^21^. Mast cells and inflammatory infiltrates were identified on toluidine blue and hematoxylin and eosin staining slides, respectively.

Microscopic analysis at 400X magnification was conducted, and results were reported semi-quantitatively using a cross scale (+, ++, +++), defined per high-power field as follows:

- Mast cells: -: 0-1; +: 2-5; ++: 6-10; +++: > 10 cells per high-power field, and
- inflammatory infiltrate: -: 0-10; +; 11-30; ++; 31-60; +++: > 60 cells per high-power field.

The thickness of the epidermis (excluding the stratum corneum) and the stratum corneum were quantified in hematoxylin and eosin-stained tissues. Collagen quantification was performed in Masson's trichrome-stained samples.

The stained tissues were digitized using the Aperio ScanScope-CS2 (Leica Biosystems, Deer Park, USA) at 400X magnification. The .svs file was analyzed using ImageScope (Aperio). The stratum corneum thickness (μm), total intensity, and number of blue-positive pixels per mm^2^ are presented as mean ± standard deviation (SD) (n = 7).

### Quantification of tissue proteins and IL-6, IL-1β, VEGF, and TNFα

Wound samples measuring 5 mm were homogenized in 1.5 ml of lysis solution composed of phosphate-buffered saline at pH 7.2, 0.2% Triton X-100, and SIGMAFAST™ protease inhibitors (Sigma-Aldrich) using an Ultra-Turrax™ (Ika, Campinas, Brazil). This process involved four 15-second cycles. The homogenates were centrifuged at 10,000g for 20 minutes at 4 °C, after which the supernatants were collected and stored at -70 °C.

The protein concentration was determined using the BCA Protein Assay Kit™ (Bio Basic Inc., NY, USA), following the manufacturer's instructions. A standard curve was constructed by diluting bovine serum albumin and measuring protein concentration from the sample optical density values. Tissue cytokine and VEGF levels were measured using a commercial ELISA kit™ (R&D Systems, Promega, Madison, WI, USA) according to the manufacturer's recommendations. Results are expressed as mean ± SD (pg of cytokine/μg of protein), normalized to total tissue protein.

### Statistical analysis

Graphs and statistical analyses were conducted using GraphPad Prism 8™ (GraphPad Software, San Diego, California, USA). Glycemia levels were compared between groups using the Kruskal-Wallis test followed by multiple comparisons with Bonferroni correction. For wound-healing area reduction percentages and AUC, a one-way analysis of variance (ANOVA) with Sidak's multiple-comparison test was performed.

Cytokine levels (IL-6, IL-1 ß, VEGF, and TNF-α) were analyzed using the Kruskal-Wallis test with Dunn's multiple comparison post-hoc test. Normal distribution was assessed with the Kolmogorov-Smirnov test. Histological parameters -including mast cell count and inflammatory infiltrate (semi-quantitative data)- were evaluated using the chi square test, while quantitative histological parameters such as stratum corneum thickness and collagen content were analyzed using ANOVA for parametric data or the Kruskal-Wallis test for non-parametric data.

Statistical significance levels were indicated as follows: comparisons against the control group (*), and comparisons between treated groups (+). Statistical significance was defined as follows: p < 0.05 (* or +), p < 0.01 (** or ++), and p < 0.001 (*** or +++).

## Results

### Induction of streptozotocin hyperglycemic mice

Blood glucose levels in BALB/c mice were monitored over 22 days following streptozotocin treatment (table 2). The control group maintained stable glucose levels around 94.02 ± 1.46 mg/dl throughout the study. In contrast, the STZ-1 and STZ-2 groups exhibited significantly higher glycemic levels than the control group on days 8, 15, and 22 (p < 0.01).

**Table 2:** Glycemia levels (mg/dl ± SD) in BALB/c mice untreated or after one or multiple doses of streptozotocin

Day	CG	STZ-1 (1 dose)	STZ-2 (5 doses)
0	94.44 ± 6.7	91.90 ± 4.57	95.73 ± 7.61
8	91.63 ± 7.36	396.16 ± 37.70	250.64 ± 34.09
15	94.42 ± 4.42	388.54 ± 29.43	283.27 ± 17.09
22	95.59 ± 5.48	390.15 ± 13.98	330.52 ± 22.95

CG: control group; STZ-1: single dose of streptozotocin; STZ-2: multiple doses of streptozotocin

Moreover, glucose levels in the STZ-1 (single dose) group were significantly higher than in the STZ-2 (multiple dose) group across days 8, 15, and 22 (p < 0.01; table 2). For reference, mean (± SD) values were 396.16 ± 37.70 versus 250.64 ± 34.09 (day 8), 388.54 ± 29.43 versus 283.27 ± 17.09 (day 15), and 390.15 ± 13.98 versus 330.52 ± 22.95 (day 22).

Elevated glycemic levels were detected 8 days following streptozotocin treatment.

In the STZ-1 dose group, similar increases were observed at 8, 15, and 22 days post-treatment (4.3-, 4.2-, and 4.2-fold increases over baseline, respectively). In contrast, multiple low-dose injections of streptozotocin induced a delayed but progressively increasing state of hyperglycemia (2.6, 3.0, and 3.5 times higher than basal values).

We included all streptozotocin-induced groups after 15 days of treatment because, on day 8, the multi-dose group had not yet exceeded the hyperglycemic threshold of 250 mg/dl for all mice, and this threshold was not maintained for at least two consecutive weeks.

### Wound closure

*Untreated hyperglycemic versus normoglycemic mice.*Figure 1 illustrates the wound-healing outcomes in control mice (untreated) on days 1, 5, and 10. The untreated hyperglycemic groups (STZ-1 and STZ-2) exhibited notably slower wound closure rates than the untreated normoglycemic mice in the control groups on days 5, 8, and 10 (p < 0.01) (figure 2). Additionally, the AUC (mm2 per days) values for the untreated hyperglycemic mice were significantly greater than those for the normoglycemic mice (control groups) (p < 0.05). This observation reinforces the link between hyperglycemia and slowed wound healing.

**Figure 1:**
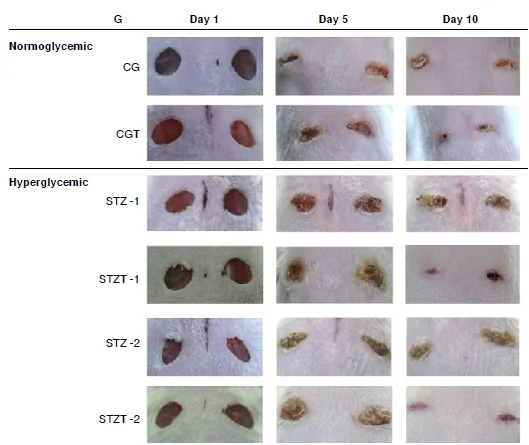
Photographs of the wound lesions on days 1, 5, and 10 in untreated and Fitostimoline™ treated BALB/c mice. Wound lesions were performed on normoglycemic and hyperglycemic (STZ-1 and STZ-2) mice as described in materials and methods. The groups treated with Fitostimoline™ included CG**T,** STZ**T**-1, and STZ**T**-2.

**Figure 2:**
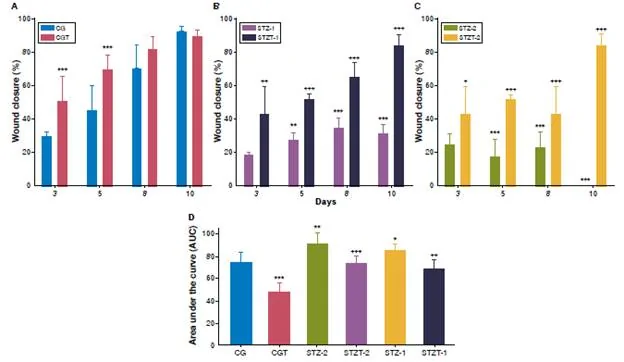
Wound healing after Fitostimoline™ treatment. Percentage reduction in lesion area on days 3, 5, 8, and 10 (ac), as well as area under the curve for lesion progression over ten days (d). The groups studied include normoglycemic and hyperglycemic STZ-1 (single dose of STZ) and STZ-2 (multiple doses of STZ) (n = 7 mice per group). The groups treated with Fitostimoline™ were CG**T,** STZ**T**-1, and STZ**T**-2. Statistical analysis was conducted using a one-way ANOVA with Sidak's multiple comparison test. Significance levels relative to the control group are indicated by an asterisk (*), while comparisons among Fitostimoline™-treated (T) groups are marked with a cross (+). Statistical significance is indicated as p < 0.05 (*, +), p < 0.01 (**, ++), and p < 0.001(***, +++).

*Fitostimoline™-treated versus untreated.* The macroscopic effects of *T. vulgare* extract treatment on wound healing outcomes on days 1, 5, and 10 are shown in figure 1. After treatment, all groups demonstrated greater wound closure than the *non-Triticum vulgare* extract treated controls (p < 0.05, figure 2a-c).

In the normoglycemic group, the wound closure percentages at 3, 5, 8, and 10 days were 50.45%, 69.84%, 82.12%, and 82.37%, respectively and differed statistically from the untreated control group on day 5 (p < 0.001; figure 2a). In the hyperglycemic mice, the wound closure percentages on the days 3, 5, 8 and 10 were 43.09%, 51.78%, 65.09%, and 84.14% for the streptozotocin treated with *T. vulgare* extract (STZT)-1 group, and 43.12%, 51.78%, 42.09%, and 84.14% for the STZT-2 group (figure 2b, c).

Within STZT-2, Kruskal-Wallis tests across days 3-8 showed no significant differences (Dunn, p > 0.05), whereas day 10 differed from day 8 (p < 0.05), indicating a late acceleration in closure. Consequently, the AUC in the *T. vulgare* extract-treated groups (control group treated, STZT-1 group, and STZT-2 group) exhibited lower values compared to their respective untreated groups (control group, STZ-1, and STZ-2); 74.24 ± 8.92 versus 48.41 ± 7.47, 85.67 ± 4.73 versus 69.07 ± 7.72, and 90.92 ± 9.60 versus 73.36 ± 6.63, respectively (p < 0.05).

### Histological analysis

*Untreated hyperglycemic versus normoglycemic mice.* Mast-cell counts were statistically similar between untreated hyperglycemic and normoglycemic mice (p > 0.05). In contrast, inflammatory cell infiltration was significantly higher in the hyperglycemic group (p < 0.05). The stratum corneum was thicker in hyperglycemic mice: control group: 76.86 ± 6.56 μm; STZ-2: 86.20 ± 2.79 μm; STZ-1: 92.57 ± 8.57 um, with both streptozotocin groups greater than control groups (p < 0.05) (table 3, figure 3). Quantitative Masson's trichrome analysis showed no significant differences in collagen deposition among untreated groups: control group: 8.10 ± 0.36; STZ-2: 8.07 ± 1.06; STZ-1: 8.07 ± 0.43 (p > 0.05) (table 3, figure 3).

**Table 3:** Histological parameters in normoglycemic and hyperglycemic BALB/c mice after Fitostimoline™ treatment. The groups treated with Fitostimoline™ were CGT, STZT-1, and STZT-2. The intensities of mast cell and inflammatory infiltrate are expressed on a scale of one to three crosses. The number of mice (n = 7) at each cross level is presented for each experimental group.

Staining	Toluidine blue				Hematoxylin and eosin			
	Histological parameter							
Experimental group	Mast cells			SL	Inflammatory infiltrate			SL
Number of crosses	+++	++	+		+++	++	+	
CG	1	6	0		2	4	1	
CGT	0	2	5	*	0	1	6	*
STZ-2	3	3	1		2	5	0	
STZT-2	1	1	5	+	0	2	5	
STZ-1	4	2	1		4	2	1	
STZT-1	0	2	5	+	0	2	5	+

STZ: streptozotocin; CG: control mice untreated; STZ-1: hyperglycemic by a single dose of STZ-1; and STZ-2: by multiple doses of STZ

SL: significance level with the control group is marked with an asterisk (*), and between treated (T) groups is marked with a cross (+). The statistical significance was p < 0.05 (*, +), p < 0.01 (**, ++), and p < 0.001(***, +++).

**Figure 3:**
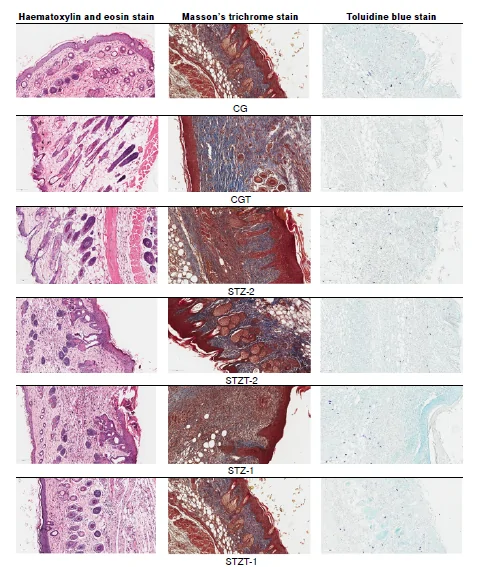
Microphotographs documenting skin tissues of mice after using various histological staining techniques. The groups studied include CG (normal group), CG**T** (plus Fitostimoline™), STZ-1 (single dose of STZ), STZ-2 (multiple doses of STZ), STZ**T**-1 (plus Fitostimoline™), and STZ**T**-2 (plus Fitostimoline™)

CG: control group untreated; CGT: control group treated; STZ-1: hyperglycemic by a single dose of STZ-1; STZ-2: hyperglycemic by multiple doses of STZ; STZT-1: hyperglycemic by a single dose of STZ-1 treated; STZ-2: hyperglycemic by multiple doses of STZ treated.

*Fitostimoline™-treated versus untreated.* Topical *T. vulgare* extract reduced inflammatory features and modulated tissue structure relative to the corresponding untreated groups. Stratum corneum thickness decreased after treatment:

- control group treated: 61.46 ± 4.93 um versus control group: 76.86 ± 6.56 μm;
- STZT-2: 66.84 ± 2.50 μm versus STZ-2: 86.20 ± 2.79 μm;
- STZT-1: 59.18 ± 9.26 μm versus STZ-1: 92.57 ± 8.57 μm (all p < 0.05).

Collagen deposition increased with *T. vulgare* extract in the control group treated (8.99 ± 0.16 versus 8.10 ± 0.36; p < 0.05) and STZT-1 (8.99 ± 0.21 versus 8.07 ± 0.43; p < 0.05). In STZT-2, collagen was numerically higher than in STZ-2 (8.66 ± 0.85 versus 8.07 ± 1.06), but this difference did not reach statistical significance. In parallel, *T. vulgare* extract-treated tissues showed lower mast-cell counts and reduced cellular infiltrates compared with their untreated counterparts (p < 0.05) (table 3, figure 3).

### Tissue levels of IL-6, IL-1β, VEGF, and TNF-α

The tissue levels of IL-6 in hyperglycemic mice (STZ-1 and STZ-2) increased significantly from 0.29 ± 0.12 pg/μg of protein in normoglycemic controls to 0.65 ± 0.25 pg/μg of protein and 0.86 ± 0.36 pg/μg of protein, respectively (figure 4a). Similarly, IL-1 ß rose from 0.21 ± 0.08 pg/μg of protein in controls to 0.67 ± 0.23 pg/μg of protein in STZ-1 and 0.64 ± 0.20 pg/μg of protein in STZ-2 (p < 0.05) (figure 4b).

**Figure 4:**
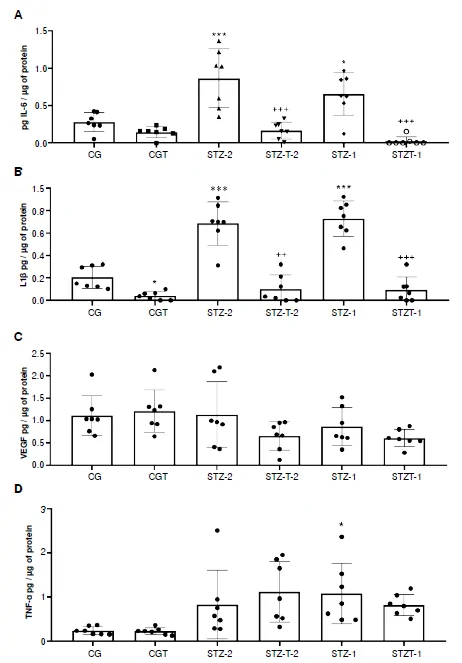
Cytokine levels after Fitostimoline™ treatment. Cytokine levels after Fitostimoline™ treatment. IL-6 (a), IL-1β (b), VEGF (c), and TNF-α (d) in lesion homogenates (pg cytokine/μg protein) in BALB/c mice. The groups studied include normoglycemic and hyperglycemic STZ-1 (single dose of STZ) and STZ-2 (multiple doses of STZ) (n = 7 in each group). The groups treated with Fitostimoline™ were CGT, STZ**T**-1, and STZ**T**-2. CG. Kruskal-Wallis tests with Dunn's multiple comparison post-hoc tests were performed as a statistical test. Significance levels for comparison with the normoglycemic group were p < 0.05 (*), p < 0.01 (**), and p < 0.001 (***); comparisons between STZ-1 versus STZ**T**-1 and STZ-2 versus STZ**T**-2 were: p < 0.05 (+), p < 0.01 (++), and p < 0.001 (+++).

CG: control group untreated; CGT: control group treated; STZ-1: hyperglycemic by a single dose of STZ-1; STZ-2: hyperglycemic by multiple doses of STZ

TNF-α also increased from 0.24 ± 0.07 pg/μg of protein in controls to 0.89 ± 0.58 pg/µg in STZ-1 (p < 0.05), whereas STZ-2 showed a non-significant increase (0.81 ± 0.72 pg/µg; p > 0.05) (figure 4d). For VEGF, there were only modest changes, from 1.02 ± 0.45 pg/µg of protein in controls to 0.62 ± 0.18 pg/µg of protein in STZ‑1 and 1.14 ± 0.68 pg/µg of protein in STZ‑2 (figure 4c).

*Fitostimoline™-treated versus untreated.* After 10 days post-treatment, *T. vulgare* extract significantly reduced IL-6 levels in hyperglycemic mice to 0.066 ± 0.10 in STZT-1, to 0.166 ± 0.103 in STZT-2 (p < 0.001), and to 0.143 ± 0.067 in normoglycemic mice (p > 0.05) (figure 4a). A similar outcome was observed for IL-1 ß, with values decreasing to 0.094 ± 0.105 in STZT-1, 0.103 ± 0.116 in STZT-2 (p < 0.01), and 0.040 ± 0.035 in normoglycemics (p < 0.05) (figure 4b).

TNF-a levels were similar between treated and untreated counterparts: STZ-1 (0.89 ± 0.58 versus 0.82 ± 0.22), STZ-2 (0.81 ± 0.72 versus 1.20 ± 0.53), and normoglycemic (control group versus control group treated; 0.24 ± 0.09 vs 0.23 ± 0.07 pg/μg of protein); all p > 0.05 (figure 4d).

VEGF showed no statistically significant changes with *T. vulgare* extract in any group (STZT-1: 0.625 ± 0.180; STZT-2: 0.661 ± 0.290; normoglycemic: 1.214 ± 0.434; p > 0.05) (figure 4c).

## Discussion

The efficacy of *T. vulgare* extract in wound healing has been amply demonstrated in normoglycemic cases; however, its efficacy in hyperglycemic conditions, such as diabetic patients, remains limited. A recent clinical study demonstrates the efficacy and safety of *T. vulgare* extract hydrogel and *T. vulgare* extract plus gauze in patients with diabetic foot ulcers ^23^. We also demonstrated its efficacy in our hyperglycemic mouse model and identified several aspects of the action mechanisms of this extract.

In our work, we induced hyperglycemia in mice, achieving values greater than 250 mg/dl, using a single high dose or multiple low doses of streptozotocin, as widely documented ^24^^,^^25^. Streptozotocin is commonly used to induce diabetes in rodents through intraperitoneal injection, typically prepared in citrate buffer at pH 4.5. In mice, a single high dose induces rapid ß-cell necrosis, resulting in blood glucose levels exceeding 300 mg/dl. In rats, single doses of 35-65 mg/kg can produce blood glucose levels ranging from 300 to 500 mg/dl, effectively modelling type 1 diabetes ^26^^,^^27^. Over time, pancreatic islet regeneration can occur, thereby reducing hyperglycemia.

Giving multiple low doses of streptozotocin causes sublethal damage to ß-cells, leading to a delayed but progressive increase in hyperglycemia and insulitis in mice and rats ^18^^,^^28^. This method mimics the autoimmune ß-cell destruction seen in type 1 diabetes, with blood glucose gradually rising above 250 mg/dl. When a low-dose of streptozotocin is combined with a high-fat diet, it speeds up ß-cell stress and models advanced type 2 diabetes.

In this model, rodents develop steady hyperglycemia (250-350 mg/dl) without notable weight loss ^29^^,^^30^. Streptozotocin enters ß-cells via the glucose transporter 2 (GLUT2) because of its structural similarity to glucose, thereby causing DNA damage, oxidative stress, and cell death ^31^.

Our results demonstrate that *T. vulgare* extract treatment effectively reduces wound size in both normoglycemic and hyperglycemic mice. In our model, this extract accelerated closure (lower AUC) and increased collagen in specific groups. The effects of *T. vulgare* extract have been studied in clinical trials and experimental models ^14^^,^^16^^,^^32^. The efficacy and tolerability of topical application of this extract in different vehicles, such as cream, impregnated gauze, foam, hydrogel, and dressing gel, have been demonstrated in diverse dermatological lesions, generally in non-diabetic patients, including second-degree superficial cutaneous burns, superficial lesions, cutaneous sores, and ulcers ^16^. Consistently, *T. vulgare* extract accelerates scratch closure in HaCaT and NIH-3T3 cells and improves wound repair in excisional models ^14^^,^^32^^,^^33^. In our data, the positive effect of *T. vulgare* extract appeared on days 3 and 5 in normoglycemic mice and on all days in hyperglycemic mice.

Histological analysis after 10 days revealed that mast cell counts were comparable between normal and hyperglycemic mice but decreased with *T. vulgare* extract. Mast cells play crucial roles in allergic reactions, immune defense, and inflammation -such as in chronic wounds and autoimmune disorders- and their role in type 1 diabetes remains unclear.

In some animal models, mast cell accumulation is harmful, whereas in others, their depletion is associated with type 1 diabetes development ^34^^,^^35^. In humans, mast cells rapidly migrate to wounds, releasing molecules that promote chemotaxis and adhesion. Their numbers drop after 6 hours, then peak at 10 days -supporting fibroblast activity, angiogenesis, and myofibroblast transformation-. When stimuli cease, they return to baseline levels. Studies suggest that mast cell accumulation can worsen inflammation and autoimmunity in type 1 diabetes models, leading to pancreatic damage and disease progression ^36^.

Skin cell infiltration increased in hyperglycemic mice; however, after *T. vulgare* extract treatment, it decreased. Similar to our model, skin from some streptozotocin-diabetic models exhibited increased stratum corneum layers and higher levels of inflammatory cells ^37^. These changes were associated with decreased keratinocyte numbers, increased inflammatory cells - particularly neutrophils-, and increased nitric oxide levels, as well as increased protein and lipid peroxide formation. The effects of *T. vulgare* extract on the skin, especially the reduction in inflammatory cell infiltration, are positive. Our results align with earlier research on *T. vulgare* extracts, which demonstrate antioxidant and anti-inflammatory effects, resulting in reduced levels of nitric oxide, IL-6, TNF-a, and prostaglandin E2 in BV2 microglial cells ^32^^,^^38^.

No significant differences in collagen deposition were observed among the untreated groups. Similar results were observed in streptozotocin-diabetic rats, in which fibroblast and collagen content in diabetic skin were comparable to those in non-diabetic controls ^39^. The lack of difference may relate to the staining method (Masson's trichrome), which does not distinguish collagen type III from type I. Type III is produced early in granulation tissue, whereas type I predominates later to increase wound strength. Diabetic rats can exhibit an imbalance between collagen synthesis and degradation, resulting in impaired contraction and reduced tensile strength ^40^.

An essential aspect of our study was that, after ten days of *T. vulgare* extract treatment, a higher number of blue pixels per mm^2^ was observed in both hyperglycemic and normoglycemic mice. The ability of *T. vulgare* extract to promote collagen expression has been directly or indirectly demonstrated in other studies; this extract promotes fibroblast growth and keratinocyte migration ^13^^,^^15^^,^^32^. The migration and closure of keratinocytes and human dermal fibroblasts were correlated with the modulation of matrix metalloproteinase-2/9, collagen I, elastin, integrin aV, aquaporin-3, fibronectin, actin polymerisation, and hyaluronan synthase-2 ^14^.

The decrease in tissue IL-6 and IL-1 after *T. vulgare* extract treatment was a notable finding, as dysregulation of IL-6 and IL-1 secretion -including spontaneous release of these cytokines by monocytes from type 1 diabetes patients- is commonly observed in various diabetic complications ^41^^,^^42^^,^^43^.

An experiment involving hyperglycemic C57BL/6 mice indicated a link between prolonged wound healing and increased expression of IL-6 and IL-6 receptor a (IL-6Rα) proteins in the wounds ^44^. On the other hand, IL-1 increases after tissue injury, promoting neutrophil recruitment, inducing fibroblast and keratinocyte proliferation, extracellular matrix protein production and degradation, fibroblast chemotaxis, and stimulation of TGF-α, keratinocyte growth factor, granulocyte-macrophage colony-stimulating, and VEGF production, a critical factor in neovascularization ^45^.

In contrast, in diabetic patients, sustained, elevated IL-1 levels are associated with type 1 diabetes and can serve as a marker to initiate therapeutic approaches targeting IL-1 ß modulation. Similar to *T. vulgare* extract topical treatment, other compounds such as nesfatin-1 (anorexigenic peptide secreted by peripheral tissues, central and peripheral nervous system), curcumin (a plant-derived metabolite from *Curcuma longa),* celecoxib (a nonsteroidal anti-inflammatory drug), and propolis (a natural bee product) have been used topically in streptozotocin-diabetic experimental models ^46^. Our study observed no differences in VEGF levels among the experimental groups.

However, Nakagawa *et al.*^47^ described a biphasic pattern of VEGF-A expression in diabetes with an initial increase followed by a decrease in later stages, possibly due to the loss of VEGF-A-producing cells ^47^. Okizaki *et al.*^48^ found that VEGF receptor type 1 signaling prevents delayed wound healing in diabetes by attenuating IL-1 ß production by recruiting macrophages ^48^. The increased TNF-α levels in STZ-1 indicate a persistent inflammatory state.

Huang *et al.*^49^ found that chronic inflammation and macrophage accumulation are standard features in diabetic wounds, possibly due to hyperglycemia's activation of the notch signalling pathway ^49^. Tsuchida *et al.*^50^ demonstrated that TNF-α suppression can significantly reduce inflammation and tissue damage in diabetic nephropathy. These findings underscore the complex interplay between angiogenesis and inflammation in diabetes, in which decreased VEGF levels may be associated with persistent inflammation, as evidenced by elevated TNF-α levels ^50^.

In conclusion, this study demonstrates that topical treatment with *T. vulgare* extract improves wound healing in normoglycemic and streptozotocin-induced hyperglycemic mice. The treatment accelerated wound closure, reduced inflammatory markers, and promoted collagen deposition. These effects were observed even in the hyperglycemic state, suggesting that *T. vulgare* extract may be a promising therapeutic option for enhancing wound healing in diabetic conditions. However, further research is needed to fully elucidate the mechanisms of action and translate these findings into clinical applications in diabetic wound care.
